# Regrouping in Dairy Ewes—Effects on Productive Performance and Specific Behavioral Traits

**DOI:** 10.3390/ani13071163

**Published:** 2023-03-25

**Authors:** Gerasimos Papakitsos, Stefania Assouad, Maria Papageorgiou, Michael Goliomytis, Maria Charismiadou, Panagiotis Simitzis

**Affiliations:** Laboratory of Animal Breeding and Husbandry, Department of Animal Science, Agricultural University of Athens, 75 Iera Odos, 11855 Athens, Greece

**Keywords:** regrouping, dairy ewe, milk yield, milk oxidative stability, heart rate, flight distance

## Abstract

**Simple Summary:**

Changes in the social environment can induce severe effects on the physiology, productivity and welfare status of dairy animals. The regrouping of dairy ewes according to age, milk production and body condition score is a common practice in sheep farms; however, data concerning its effects on productive and behavioral traits are scarce. As shown in the present study, regrouping triggered an emotional distress in ewes, since the number of bleats, heart rate and flight distance were increased immediately after regrouping. Moreover, milk yield and fat were reduced, and milk oxidation rates and the number of kick responses during milking were increased. It can be concluded that the regrouping of dairy ewes could negatively influence productive and behavioral parameters until the reestablishment of social relationships.

**Abstract:**

The regrouping of ruminants is a common practice in dairy farms and is targeting at the improvement of milk production efficiency. However, changing the established hierarchy in a group affects both productivity and behavioral attributes. The aim of the present study was therefore to examine the possible effects of regrouping on milk yield, composition and several behavioral indices in dairy ewes. The experimental period was divided into two sub-periods of 20 days each. During the first period, 30 Chios ewes were used, while 15 Chios and 15 Karagouniko ewes were mixed during the second period. Milk yield, composition, oxidative stability, flow rate and number of kick responses during milking were recorded for each ewe. An isolation—flight distance test was also performed on a weekly basis. As indicated, the parameters recorded during the behavioral tests, such as the number of bleats, heart rate and flight distance, and the milk oxidation rate and number of kick responses were significantly increased immediately after regrouping compared to the following days during both sub-periods (*p* < 0.05). Moreover, milk yield was reduced in the second sub-period by 8.61% (*p* < 0.05). It can be concluded that regrouping could negatively influence productive and behavioral traits, and the effect was more pronounced when sheep of different breeds were mixed.

## 1. Introduction

Farm animals are social species that ideally live in socially stable groups. Belonging in a group and engaging in animal-to-animal interactive behaviors is experienced as positive and rewarding by the animals [1]. As members of a group, animals experience cohesion and safety, synchronize their behavioral repertoire, display social affiliative behaviors [2,3], cooperation and pro-social behaviors [4]. Moreover, the sense of belonging to a group appears to provide a buffering effect on stress perception [3,5].

Regrouping is a common management practice in livestock farming, especially for the intensively housed animals. Animals are being regrouped or regrouped and relocated not only throughout their different productive stages, but also during transport and slaughter. Regrouping disturbs the group’s complex social stability and leads to social stress for both newcomers and the individuals of the group [6]. The social order is being disrupted and the new members are usually encountered with aggression. Due to the emotional contagion of behaviors in social species, the stress and agonistic behaviors can be spread amongst the whole group [5]. The results are more severe for the low-ranked animals, especially when regrouping leads to crowding and competition for resources. It is a social stressor that can cause physiological and behavioral changes and contribute to disease in farm animals [7]. Not only is the welfare status impaired, but the production is also impaired in both quality and quantity, as shown in cattle [8,9,10], goats [11] and sheep [12]. Thus, in addition to being a risk factor for the animals’ welfare and health, regrouping can be a threat to the economical profit of the farmer.

As it is a standard procedure in intensive dairy farming systems, regrouping has been extensively studied in cattle. Regrouping affects the behavior of dairy cows [9,13], especially when combined with relocation [13]. The duration of allogrooming, lying time [9], feeding time and rumination [9,13] is reduced during the first days and until the reestablishment of social relationships. The negative effects of regrouping are greater for the animals with a lower dominance ranking [8,14]. Milk production is also reduced [8,9,10], and the impact is higher on primiparous cows compared to multiparous ones [10]. Repeated regrouping does not seem to stress calves, since they tend to become accustomed to this procedure [15]. On the contrary, heifers do not easily become habituated and constantly express agonistic behaviors [16]. Furthermore, regrouping seems to not influence the reproductive performance of cattle [17,18]. As indicated, reduced stocking density [19] and early social contact [20] can mitigate the negative effects of regrouping.

In goat farms, neither regrouping nor the introduction of new individuals into a group are recommended since they disrupt the process of individual recognition [21] and increase aggression [11,22]. According to Fernandez et al. [11], milk production decreases in French Alpine goats when regrouping is initially applied but not after subsequent regroupings, suggesting that the breed has a high adaptation capacity to stressful situations. Regrouping in Saanen goats resulted in decreased feeding and resting times, but kid growth and survival remained unaffected [22]. Furthermore, regrouping unknown bucks has a negative impact on their welfare and also reduces their reproductive performance [23].

The literature on the effects of regrouping on sheep welfare and production is limited. Sevi et al. [12] observed that regrouping led to aggressive interactions in both the newcomer and the remaining ewes. In addition, cell immune response was suppressed, and udder health was negatively impacted. Mastitis was observed in the regrouped animals but not in the control group. Regrouping also resulted in short-term reduction of milk production and in lower protein and fat milk content. In another study, Engeldal et al. [24] examined the behavioral reactions of rams of different breeds after regrouping. In all cases, agonistic behavior was the most commonly observed. Stocking density after regrouping and the breed of the rams influenced their adaptation capacity. Pareek and Kataria [25] also observed increased oxidative stress to Magra sheep during regrouping and transportation, especially under extreme humid and hot conditions, and suggested that supplementation with antioxidants could make this management procedure less difficult for the animals.

The regrouping of dairy ewes according to age, milk production and body condition score is a common practice in sheep farms. Nonetheless, data regarding the effects of this practice on sheep productive and behavioral traits are scarce. Based on previous results in other species, it is predicted that the abrupt break of social relationships can lead to stress reactions and transitory negative effects in the productivity of sheep, a species that is characterized by its gregariousness. The primary aim of the current study was to test the short-term effects of regrouping on several welfare indices in intensively managed dairy ewes. A secondary objective with a more practical value was to determine the impact of regrouping on milk yield, composition and oxidative stability, which is a crucial parameter for the assessment of the cheese-making value of sheep milk. We further hypothesized that the effects of regrouping and relocation between animals of different breeds could vary. The aim of the present study was therefore to examine the effects of regrouping on milk yield, milk composition and several behavioral indices in two different breeds of dairy ewes. 

## 2. Materials and Methods

### 2.1. Animals

Sixty two-year-old ewes with similar body condition scores (2.5–3) which were at their second parity two months after lamb weaning (105 ± 5 days after lamb birth) were randomly selected from the flock of the experimental farm of the Agricultural University of Athens and used for the present experiment. The group comprised 45 Chios and 15 sheep of the Karagouniko breed with a mean weight of 62.1±3.5 and 53.4±2.3 kg, respectively. The ewes of the flock were mated following estrus synchronization with intravaginal progestogen sponges (Ovigest, Hipra, Amer, Spain). As a result, all births took place within a week. Before the beginning of the experiment, the ewes remained in random groups of approximately 30 individuals according to the time of their birth. The groups were thus stable for 60 days: from weaning (45th) until the 105th day after the birth of the lambs (the beginning of the experiment). The feeding regime and the handling were similar before and during the entire experimental period. All animals consumed alfalfa hay and the same concentrated basal diet (Table S1), according to their nutritional needs. The 30 regrouped ewes were housed in one pen that was different from the pen they were housed in before regrouping. All pens were identical, with a similar orientation, a covered area and troughs for feeding. In detail, the space allowance was 2 m^2^ and the trough space was 0.5 m per ewe. 

The experimental period was divided into two sub-periods of 20 days. The first period included 30 Chios ewes, while 15 Chios and 15 Karagouniko ewes were regrouped and relocated during the second sub-period. After regrouping, both groups remained stable for 20 days. Between the two sub-periods, there was an interval of ten days. The Chios ewes used in the second sub-period were not used during the first sub-period. 

### 2.2. Recorded Parameters during Milking

The ewes were milked twice per day (6:00 a.m. and 18:00 p.m) in a 12 stall milking parlor (GEA Westfalia, Düsseldorf, Germany). A pulsation ratio of 50:50 was applied: the pulsation rate was 150 cycles min^−1^ with 37.5 kPa vacuum level. Milk yield (quantity derived from the morning and afternoon), milk flow rate (mL/s) (milk yield divided by the time between attachment and detachment of the teat cup) and the number of kick responses (raising a hoof at the height of the udder) during the milking of each ewe were recorded on day 1 prior to regrouping and on days 1, 2, 3, 4, 5, 13 and 20 after regrouping. Individual milk samples were also collected during the same sampling days for the determination of milk composition and oxidative stability. 

### 2.3. Determination of Milk Composition and Oxidative Stability

Milk samples were analyzed for fat, protein, lactose, and total solids-not-fat, using a Milkoscan 133 (Foss Electric, Hillerød, Denmark) calibrated for sheep milk according to the Mojonnier method for fat, the Kjeldahl method for protein, and the polarimetric method for lactose [26]. The milk oxidative stability was evaluated by measuring the levels of malondialdehyde (MDA), a secondary lipid oxidation product formed by hydrolysis of lipid hydroperoxides. MDA concentration was determined by applying a selective, third-order derivative spectrophotometric method previously developed by Botsoglou et al. [27].

### 2.4. Behavioral Recordings during Isolation—Flight Distance Test

The dairy ewes were also subjected to a behavioral test designed for the evaluation of emotional distress: the isolation and flight distance test, carried out on the 1st, 6th, 13th and 20th day after regrouping. We selected these two types of tests since we hypothesized that the agitated ewes would display more intense stress responses (i.e., to human approach) due to the disturbance of their social hierarchy. The tests were conducted between 9:00 and 12:00 a.m. on each experimental day. Before testing, the ewes were removed to a holding pen, which they remained in for 30 min. After this adaptation period, each individual ewe was separated from the group and moved to the entrance of the test pens (ca. 7m distance). The procedure was designed to ensure that the handling protocol applied to the animals between the holding and test pen was standardized as far as possible. The ewes were randomly tested so that the treatment factor was not confounded with the order of testing. 

The isolation portion of the test consisted of social isolation in a novel environment (1 min) without any additional fear-inducing stimuli [28,29]. The test pen represented a novel environment in which the tested animal was visually isolated from other members of the group. Each pen (2 m × 2 m) had solid walls, and the floor was covered with sawdust. The average, minimum and maximum heart rate of lambs (beats per minute–bpm) was measured using the POLAR S180i heart rate monitor system (Polar Electro Oy, Helsinki, Finland) during the isolation test (1 min). This system was adapted to the ewe at the beginning of the adaptational period (30 min) in the holding pen, but it was activated just before the entry to the test pen. The system consisted of the transmitter and the receiver, which detected and displayed the heart rate, respectively. The transmitter was fitted with an elastic strap which held it firmly in the heart girth (the area around body behind front legs) of each lamb. The electrode areas were moisturized with a gel appropriate for ultrasonic and electrical transmission (Ultrasound, Milan, Italy) and put against the ewes’ skin. Vocalizations (bleats) were also recorded during the isolation test as they can reflect the emotional state of dairy ewes.

The person who had introduced the ewe into the isolation pen then remotely opened the front gate from behind a hessian screen at the back of the pen. As the ewe walked forward out of the cage, a second person appeared at the opposite end of the 20 m race and walked towards the ewe at a speed of approximately 1 m/s. The flight distance was defined as the interval between the human and the sheep when the ewe began its run past the approaching experimenter; in other words, the distance a human can approach a sheep before it retreats [30].

### 2.5. Statistical Analysis

All data, apart from the behavioral parameters, were subjected to a repeated measures analysis of variance using the MIXED procedure on SAS software, using the sampling day before and after regrouping as the fixed factor. Results are presented as the least square means ± the standard error of mean. The effect of the breed was also examined in the second sub-period; however, it was not found to be significant, and it was excluded from the model. Behavioral data, i.e., kick responses, number of vocalizations and flight distance, did not follow a normal distribution and were subjected to a non-parametric ANOVA with the NPAR1WAY procedure on the SAS software. The results are presented as medians. Significant differences were tested at a 0.05 significance level.

## 3. Results

### 3.1. Regrouping of Ewes Belonging to the Same Breed (Chios)

Milk yield and composition were generally not significantly influenced by regrouping (Table 1). In detail, the values for milk protein, lactose and total solids-non-fat were similar on day −1 vs 1, 2, 3, 4, 5, 13 and 20 (*p* > 0.05). Only the milk fat (%) appeared to decrease after the 4^th^ day of regrouping (*p* < 0.05). Milk oxidation rates were increased immediately after regrouping, but the milk MDA content returned to its initial values on day 2 after regrouping (Table 1). No significant effect of regrouping on milk flow rate was observed (*p* > 0.05), while the number of kick responses was increased on day 1, 2 and 3 after regrouping (*p* < 0.05)

As indicated in Table 2, the average, maximum and minimum heart rate, number of vocalizations and flight distance were significantly increased on the first day after regrouping (*p* < 0.05). The values of the aforementioned parameters were decreased on day 6 and remained at similar levels on day 13 and 20 (*p* > 0.05).

### 3.2. Regrouping of Ewes Belonging to Different Breeds (Chios and Karagouniko)

In contrast with the findings of the first sub-period, when only one breed was used, the milk yield was significantly decreased as an effect of regrouping on day 1 (Table 3; *p* < 0.05). The milk protein, lactose and total solids-non-fat were not significantly affected by regrouping (*p* > 0.05). The milk fat percentage (%) was decreased after the third day of regrouping (*p* < 0.05). Milk oxidation rates were increased immediately after regrouping, but milk MDA content returned to its initial values on day 2 after regrouping (Table 3). No significant effect of regrouping on the milk flow rate and the number of kick responses was observed (*p* > 0.05).

As indicated in Table 4, the average, maximum and minimum heart rate were significantly increased during the first and sixth day after regrouping, but they decreased afterwards on day 13 and 20 (*p* < 0.05). The flight distance was also increased on day 1 but was decreased on day 6 and remained at similar levels on day 13 and 20. Interaction of day by breed was not significant for the examined parameters during the regrouping of different sheep breeds (*p* > 0.05) with the exception of number of vocalizations (Tables S2 and S3), indicating that both breeds in general responded similarly to the examined treatment. Chios ewes reduced their vocalizations in the days after regrouping, whereas no significant differences were observed in Karagouniko ewes. At the same time, milk fat and milk flow rate (Table S2) had significantly higher values and the number of vocalizations and flight distance (Table S3) had significantly lower values in Karagouniko than Chios ewes (*p* < 0.001). 

## 4. Discussion

As indicated, the milk yield was not affected when ewes of a similar breed were regrouped (Table 1), while the regrouping of different breeds of sheep resulted in decreased milk production during the first day (Table 3). Apart from fat percentage, the milk flow rate and composition were not influenced by regrouping (Table 1 and Table 3). However, the milk flow rate was lower in Chios compared to the Karagouniko breed, a finding that was already demonstrated in a previous study [28]. The value (%) for milk fat was reduced, while the milk oxidation rates and number of kick responses were increased on the first day after regrouping (Table 1 and Table 3). Several researchers have pointed out a short-term decrease in milk production in dairy cattle that were regrouped, possibly as a result of increased competitive interactions [8,9,10,31]. A similar effect was observed in goats [11] and sheep [12]. The reduction in milk fat percentage as an effect of regrouping was also demonstrated by Sevi et al. [12] in Comisana ewes. The milk fat percentage was also higher in Karagouniko than Chios ewes, which was expected from the literature [32]. Milk MDA values, which could serve as an index of oxidative stress, were also increased on the first day after regrouping. This finding is in agreement with that of Pareek and Kataria [25], who pointed out that stressors such as regrouping could induce oxidative stress in Magra sheep. The number of kick responses was also increased as an effect of regrouping, possibly indicating the emotional distress experienced by the ewes due to the disturbance of their social hierarchy. In general, the number of flinch, step and kick responses are associated with low productivity and could be interpreted as a sign of agitation in dairy cattle [33,34]. 

Behavioral reactivity tests were applied to assess differences among the displayed behaviors of the animals, allowing producers to identify favorable management practices [35]. Locomotory activity (i.e., flight distance) and the vocalizations recorded during these types of tests can be regarded as accurate indicators for the assessment of distress response and behavioral reactivity in sheep [28,29]. The observed sheep behavior can indicate welfare or management problems due to husbandry procedures and can provide clues to the types of situations that might elicit a stress response [36,37] and can lead to an economic impact on sheep production, since highly reactive animals generally display reduced productivity [35]. As shown in the present study (Table 2 and Table 4), the average, maximum and minimum heart rate, the number of vocalizations and the flight distance were significantly increased on the first day after regrouping, indicating great emotional distress in the regrouped ewes. In general, mixing unfamiliar animals leads to increased aggressive interactions (threats, butting, etc.) and reduced lying and feeding times in cows [9,38], goats [11] and sheep [12]. Moreover, Chios ewes bleated more and had a shorter flight distance compared to the Karagouniko ewes, possibly as a result of differences in their temperament and the extent to which they fear people, as was already pointed out by Simitzis et al. [28]. 

Future research should aim to study possible means of mitigating the negative impacts of regrouping. Studies in dairy cattle have shown that the negative impacts of regrouping on animal behavior and production are more severe when combined with increased stocking density after regrouping periods. Talebi et al. [19] observed a decrease in competitive behavior and an increase in lying time in Holstein cows when stocking density was decreased for the regrouping practice. Fu et al. [39] also observed that the welfare of pigs is more impaired in high stocking densities after regrouping. Thus, the relationship between regrouping and stocking density should be also studied in dairy ewes. 

At the same time, attention should be paid to examining the early social behavior development of the lamb. The early social contact of the calf leads to an improved response to the first regrouping [20], and early life socialization in piglets reduces the post-regrouping stress [40]. In addition, according to Foris et al. [41], the familiarity of cows influences the social network after regrouping and has a social buffering effect since after regrouping, cows prefer to express feeding and affiliative behaviors with the group individuals that they already knew during the pre-regrouping period. The impact of regrouping on the social network of the sheep should be studied in more detail. It should be also examined whether it is more severe for the low-ranked animals, as has been concluded for cattle [8,14]. In our study, negative impacts on production and welfare were highlighted, but the difference between low- and high-ranked animals was not observed since the social network of each experimental group was not studied in advance. This information is important because avoiding mixing ewes with very high and very low dominance rankings could, in practice, alleviate the negative effects of regrouping. Furthermore, in cattle, the impacts of regrouping are greater for primiparous cows compared to multiparous ones [10]. Therefore, another management practice that can be evaluated as a way of mitigating the negative effects of regrouping could be the avoidance of mixing multiparous and primiparous ewes or avoiding regrouping primiparous ewes in general. Furthermore, feed enrichment with antioxidants could also alleviate the negative impact of oxidative stress due to the regrouping procedure. Finally, according to Sevi et al. [12], the animals that are submitted to regrouping and relocation have a lower milk yield and milk fat content that the animals that experienced only regrouping. 

In our study, milk yield was reduced when ewes of Chios and Karagouniko breeds were mixed, but not when only ewes of Chios were re-grouped. However, interaction of day by breed was not significant for the examined parameters with the exception of vocalizations, indicating that the performance and behavioral traits were similarly affected in the Chios and Karagouniko ewes. In contrast, Engeldal et al. [24] observed that rams of different breeds reacted differently to regrouping with respect to behavioral responses, reproductive performance and time needed for adaptation.

As far as the authors are concerned, this is the first study that investigates the effects of regrouping as a management procedure on milk oxidation rate, and also on several behavioral traits recorded by the implementation of an isolation—flight distance test in dairy ewes. We concluded that regrouping triggers oxidative stress in sheep, which is indicated as a short-term increase in milk MDA content after regrouping, but also induces a welfare impairment, demonstrated the increased values of heart rate, number of vocalizations and flight distance. Therefore, regrouping has a moderate but limited negative impact on the performance and welfare of ewes. 

## 5. Conclusions

The regrouping of dairy ewes according to age, milk production and body condition score is a common practice in sheep farms for achieving homogenous groups in terms of milk yield or stage of lactation. As can be concluded by the present study, the regrouping of dairy ewes could have a transitory influence on productive and welfare traits, and the effect was more pronounced when sheep of different breeds were mixed. 

## Figures and Tables

**Table 1:** Effect of regrouping on milk yield, composition, oxidative stability, flow rate and kick responses in Chios ewes.

Examined Parameter	Day before and after Regrouping ^1^								
	−1	1	2	3	4	5	13	20	SEM
Daily milk yield (mL)	1194	1169	1213	1274	1159	1272	1173	1091	81
Milk fat (%)	5.20 ^a^	5.16 ^a^	5.19 ^a^	4.98 ^a^	4.63 ^b^	4.70 ^b^	4.66 ^b^	4.71 ^b^	0.17
Milk protein (%)	4.97	5.01	4.91	5.06	5.11	5.08	5.00	5.02	0.07
Milk lactose (%)	4.68	4.69	4.73	4.61	4.69	4.63	4.63	4.60	0.04
Milk total solids-not-fat (%)	10.20	10.24	10.19	10.22	10.33	10.25	10.18	10.16	0.08
Milk malondialdehyde (MDA) levels (ng/mL)	10.31 ^a^	12.64 ^b^	10.69 ^a^	9.26 ^a^	10.45 ^a^	9.13 ^a^	10.95 ^a^	10.17 ^a^	0.51
Milk flow rate (mL/s)	7.42	8.41	8.06	7.40	6.43	7.03	7.23	6.41	0.50
Kick responses ^2^	0 (1) ^a^	1 (2) ^b^	1 (2) ^b^	1 (3) ^b^	1 (2) ^a^	0 (1) ^a^	0 (2) ^a^	0 (2) ^a^	

^1^ Day −1: 1 day before, and Days 1, 2, 3, 4, 5, 13 and 20: 1st, 2nd, 3rd, 4th, 5th, 13th and 20th day after regrouping. ^2^ Presented as medians and interquartile range in parenthesis. ^a,b^ Means or medians within a row with different superscripts are significantly different (*p* < 0.05)

**Table 2:** Effect of regrouping on the average, minimum and maximum heart rate (beats/min), the number of vocalizations per min, and the flight distance (m) during the isolation—flight distance test in Chios ewes.

Examined Parameter	Day after Regrouping ^1^				
	1	6	13	20	SEM
Average heart rate	145 ^a^	124 ^b^	130 ^b^	127 ^b^	3.7
Maximum heart rate	202 ^a^	170 ^b^	165 ^b^	153 ^b^	6.86
Minimum heart rate	116 ^a^	100 ^b^	104 ^b^	105 ^b^	2.49
Number of vocalizations ^2^	7 (7) ^a^	3 (5) ^b^	2 (6) ^b^	3 (6) ^b^	
Flight distance (m) ^2^	2 (2) ^a^	1.5 (1.5) ^b^	1 (1.5) ^b^	1 (0.5) ^b^	

^1^ Day 1, 6, 13 and 20: 1st, 6th, 13th and 20th day after regrouping. ^2^ Presented as medians and interquartile range in parenthesis. ^a,b^ Means or medians within a row with different superscripts are significantly different (*p* < 0.05).

**Table 3:** Regrouping of different sheep breeds and effects on milk yield, composition, oxidative stability, flow rate and kick responses.

Examined Parameter	Day before and after Regrouping ^1^								
	−1	1	2	3	4	5	13	20	SEM
Daily milk yield (ml)	1288 ^a^	1177 ^b^	1298 ^a^	1274 ^a^	1212 ^a^	1240 ^a^	1167 ^b^	1100 ^b^	74
Milk fat (%)	4.83 ^a^	4.83 ^a^	4.81 ^a^	4.46 ^b^	4.69 ^b^	4.63 ^b^	4.61 ^b^	4.57b	0.11
Milk protein (%)	5.13	5.23	5.20	5.27	5.28	5.25	5.25	5.15	0.07
Milk lactose (%)	4.70	4.62	4.67	4.66	4.61	4.65	4.65	4.64	0.04
Milk total solids-not-fat (%)	10.38	10.40	10.43	10.48	10.54	10.45	10.46	10.43	0.08
Milk malondialdehyde (MDA) levels (ng/mL)	8.41 ^a^	11.30 ^b^	8.75 ^a^	10.06 ^a^	10.16 ^a^	9.74 ^a^	10.04 ^a^	10.14 ^a^	0.36
Milk flow rate (mL/s)	8.43	7.25	8.12	7.60	7.41	8.04	7.54	7.61	0.67
Kick responses ^2^	0 (1)	0.5 (2)	0 (1)	0 (1)	0 (1)	0 (0)	0 (0)	0 (0)	

^1^ Day −1: 1 day before, and Days 1, 2, 3, 4, 5, 13 and 20: 1st, 2nd, 3rd, 4th, 5th, 13th and 20th day after regrouping. ^2^ Presented as medians and interquartile range in parenthesis. ^a,b^ Means or medians within a row with different superscripts are significantly different (*p* < 0.05).

**Table 4:** Regrouping of different sheep breeds and effects on the average, minimum and maximum heart rate (beats/min), the number of escape attempts and vocalizations per min, and the flight distance (m) during the isolation—flight distance test.

Examined Parameter	Day after Regrouping ^1^				
	1	6	13	20	SEM
Average heart rate	130 ^a^	124 ^a^	112 ^b^	112 ^b^	3.2
Maximum heart rate	180 ^a^	166 ^a^	142 ^b^	136 ^b^	5.8
Minimum heart rate	101 ^a^	98 ^a^	90 ^b^	93 ^b^	2.3
Number of vocalizations ^2^	6.5 (6) ^a^	5.5 (5) ^a^	4 (4) ^a^	4 (4) ^b^	
Flight distance (m) ^2^	2.5 (1) ^a^	1.5 (1.5) ^b^	1 (1.5) ^b^	1 (1) ^b^	

^1^ Day 1, 6, 13 and 20: 1st, 6th, 13th and 20th day after regrouping. ^2^ Presented as medians and interquartile range in parenthesis. ^a,b^ Means or medians within a row with different superscripts are significantly different (*p* < 0.05).

## Data Availability

The data presented in this study are available upon request from the corresponding author.

## Supplementary Materials

The following supporting information can be downloaded at: https://www.mdpi.com/article/10.3390/ani13071163/s1, Table S1: Composition and analysis of dairy ewes’ diet; Table S2: Effects of ewe breed on milk yield, composition, oxidative stability, flow rate and kick responses; Table S3: Effects of ewe breed on the average, minimum and maximum heart rate (beats/min), the number of escape attempts and vocalizations per min and the flight distance (m) during the isolation—flight distance test.
