# Supplementary material for: Regrouping in Dairy Ewes—Effects on Productive Performance and Specific Behavioral Traits
Source: Animals (Basel). 2023 Mar 25;13(7):1163. doi: 10.3390/ani13071163 (PMC10093312; doi:10.3390/ani13071163)
Supplement: Supplementary file 1 [file animals-13-01163-s001.zip › animals-2242406-supplementary.pdf]

animals

MDPI

Regrouping in Dairy Ewes—Effects on Productive Performance and Specific Behavioral Traits

Table S1. Composition and analysis of dairy ewes' diet.

Components (g/kg)		
Corn	465	
Wheat	120	
Soybean Meal (44%)	210	
Sunflower Meal	50	
Alfalfa Meal	30	
Wheat Bran	40	
Plant Oil	25	
Molasses	15	
Sodium Chloride (NaCl)	9	
Calcium Carbonate	18	
Monocalcium Phosphate	14	
Vitamins & Trace elements Premix*	4	
Calculated Analysis	*Concentrates*	*Alfalfa hay*
Dry Matter – DM (%)	88.0	93.5
Crude protein – CP (%)	17.0	10.2
Crude Fiber (%)	5.4	34.2
Ash (%)	8.0	7.4
Fat (%)	5.0	2.3
Calcium (%)	1.0	-
Phosphorus (%)	0.7	-
Sodium (%)	0.4	-

*Premix contained per kg: 150 mg Mg, 35 mg Mn, 50 mg Fe, 60 mg Zn, 0.8 mg Se, 0.75 mg Co, 1.25 mg I, 60 mg Se, 200 mg Mo, 15 kIU vitamin A, 2 kIU vitamin D3, 25 mg vitamin E (kIU: 1000 International Units).

Table S2. Effects of ewe breed on milk yield, composition, oxidative stability, flow rate and kick responses.

Breed	Examined Parameter	Day before and after Regrouping [1]								
		**−1**	**1**	**2**	**3**	**4**	**5**	**13**	**20**	**SEM**
Chios	Daily milk yield (mL)	1369 [a]	1272 [b]	1409 [a]	1319 [ab]	1384 [a]	1362 [a]	1309 [ab]	1209 [b]	101
	Milk fat (%)	4.35 [a]	4.40 [a]	4.52 [a]	3.89 [b]	4.33 [a]	4.23 [a]	4.11 [ab]	4.15 [ab]	0.16
	Milk protein (%)	5.06	5.21	5.19	5.21	5.22	5.19	5.07	5.09	0.10
	Milk lactose (%)	4.61	4.56	4.62	4.67	4.63	4.61	4.65	4.69	0.06
	Milk total solids-not-fat (%)	10.22	10.32	10.36	10.43	10.51	10.34	10.26	10.22	0.11
	Milk malondialdehyde (MDA) levels (ng/mL)	8.97 [a]	11.86 [b]	8.76 [a]	9.60 [a]	10.93 [b]	10.03 [a]	9.95 [a]	10.57 [ab]	0.49
	Milk flow rate (mL/s)	6.00	6.10	5.92	5.50	5.80	6.21	6.22	5.86	0.91
	Kick responses [2]	0 (2)	2 (2)	0 (1)	1 (1)	0 (1.5)	0 (0)	0 (1)	0 (3)	
Karagouniko	Daily milk yield (mL)	1207 [a]	1082 [b]	1186 [a]	1229 [a]	1239 [a]	1217 [a]	1025 [b]	989 [b]	108
	Milk fat (%)	5.30 [a]	5.25 [a]	5.09 [ab]	5.03 [b]	5.06 [b]	5.03 [b]	5.11 [ab]	4.99 [a]	0.17
	Milk protein (%)	5.21	5.25	5.21	5.32	5.33	5.32	5.43	5.21	0.10
	Milk lactose (%)	4.78	4.68	4.73	4.64	4.69	4.69	4.66	4.69	0.06
	Milk total solids-not-fat (%)	10.54	10.49	10.49	10.52	10.58	10.57	10.65	10.45	0.11
	Milk malondialdehyde (MDA) levels (ng/mL)	7.85 [a]	10.75 [b]	8.73 [a]	10.53 [b]	9.38 [a]	9.45 [a]	10.14 [ab]	9.72 [ab]	0.52
	Milk flow rate (mL/s)	10.86	9.09	10.32	9.69	9.02	9.86	8.86	9.36	0.98
	Kick responses [2]	0 (0)	0 (1)	0 (1)	0 (1)	0 (1)	0 (0)	0 (0)	0 (0)	
p-value		Breed	Day * Breed							
	Daily milk yield (mL)	0.176	0.157							
	Milk fat (%)	0.001	0.127							
	Milk protein (%)	0.224	0.057							
	Milk lactose (%)	0.513	0.063							
	Milk total solids-not-fat (%)	0.129	0.140							
	Milk malondialdehyde (MDA) levels (ng/mL)	0.132	0.173							
	Milk flow rate (mL/s)	0.001	0.563							
	Kick responses	0.445	0.749							

[1] Day -1: 1 day before and Days 1, 2, 3, 4, 5, 13 and 20: 1st, 2nd, 3rd, 4th, 5th, 13th and 20th day after regrouping [a,b] Means within a row with different superscripts are significantly different ($p < 0.05$) [2] Presented as medians and interquartile range in parenthesis.

Table S3. Effects of ewe breed on the average, minimum and maximum heart rate (beats/min), the number of escape attempts and vocalizations per min, and the flight distance (m) during the isolation—flight distance test.

**Breed**	**Examined Parameter**	**Day after Regrouping** [1]				
		**1**	**6**	**13**	**20**	**SEM**
Chios	Average heart rate	129 [a]	123 [a]	104 [b]	105 [b]	4.38
	Maximum heart rate	186 [a]	160 [a]	128 [b]	132 [b]	7.95
	Minimum heart rate	102 [a]	98 [a]	87 [b]	87 [b]	3.21
	Number of vocalizations [2]	10 (5) [a]	6.5 (1) [b]	5.5 (5) [bc]	4 (3) [c]	
	Flight distance (m) [2]	2 (1.5) [a]	1 (1.5) [b]	0.5 (1) [b]	0.5 (0.8) [b]	
Karagouniko	Average heart rate	131 [a]	126 [a]	120 [ab]	118 [b]	4.69
	Maximum heart rate	174 [a]	172 [a]	155 [b]	139 [b]	8.50
	Minimum heart rate	101 [a]	99 [a]	93 [b]	93 [b]	3.43
	Number of vocalizations [2]	3 (6)	2 (5.5)	3.5 (6)	3.5 (4.5)	
	Flight distance (m) [2]	3 (3) [a]	1.5 (2) [b]	1.5 (1) [b]	1 (1) [c]	
		Breed	Day * Breed			
p-value	Average heart rate	0.084	0.144			
	Maximum heart rate	0.284	0.078			
	Minimum heart rate	0.196	0.144			
	Number of vocalizations	0.001	0.001			
	Flight distance (m)	0.001	0.429			

[1] Day 1, 6, 13 and 20: 1st, 6th, 13th and 20th day after regrouping [a,b,c] Means within a row with different superscripts are significantly different ($p < 0.05$). [2] Presented as medians and interquartile range in parenthesis.
